# Retinal Vascular Density on OCT-A and Age-Related Central and Peripheral Hearing Loss in an Italian Older Population

**DOI:** 10.1093/geroni/igab046.596

**Published:** 2021-12-17

**Authors:** Rodolfo Sardone, Ilaria Bortone, Francesco Panza, Giancarlo Sborgia, Gianluigi Giuliani, Valentina Pastore, Francesco Boscia, Nicola Quaranta

**Affiliations:** 1 Population Health - Sensorial Frailty Laboratory - NATIONAL INSTITUTE IRCCS “S DE BELLIS” Research Hospital, Castellana Grotte, Puglia, Italy; 2 Institute of Clinical Physiology - National Research Council, Pisa, Toscana, Italy; 3 Gerontology Unit, National Institute of Gastroenterology "Saverio de Bellis", Research Hospital, Castellana Grotte, Bari, Italy., Castellana Grotte, Puglia, Italy; 4 Eye Clinic, Department of Basic Medical Sciences, Neuroscience and Sense Organs, University of Bari "Aldo Moro", Bari, Italy., Bari, Puglia, Italy; 5 Otolaryngology Unit, Department of Basic Medical Science, Neuroscience and Sense Organs, University of Bari Aldo Moro, Bari, Italy., Bari, Puglia, Italy

## Abstract

Age-related hearing loss (ARHL) and retinal vessel changes have both been associated to neurodegeneration/dementia, suggesting a possible link between these two conditions in older age. We analyzed data on 886 older participants (65 years+, age range: 65-92 years) in the cross-sectional population-based Salus in Apulia Study. OCT-A scan was used to measure SVD and DVD of the capillary plexi of the macula in different retinal quadrants. Peripheral ARHL was defined as >40 dB HL of PTA (0.5,1,2, and 4KHz) in the worst ear, and age-related CAPD as <50% at the SSI-ICM test in at least one ear. DVD at the whole retina and at the parafoveal quadrant were inversely associated only with age-related CAPD [OR:0.93; 95%CI: 0.88-0.96 and OR:0.94; 95 CI:0.90-0.99, respectively]. The association of retinal vascular density with age-related CAPD may bring us a further step forward in understanding the biological mechanisms underlying the links between neurodegeneration/dementia and ARHL.

